# Legumes as basic ingredients in the production of dairy‐free cheese alternatives: a review

**DOI:** 10.1002/jsfa.11502

**Published:** 2021-09-09

**Authors:** Marina Mefleh, Antonella Pasqualone, Francesco Caponio, Michele Faccia

**Affiliations:** ^1^ Department of Soil, Plant and Food Science (DISSPA) University of Bari Aldo Moro Bari Italy

**Keywords:** dairy‐free products, legume proteins, anti‐nutritional factors, vegan; sustainability, technological properties

## Abstract

Research into dairy‐free alternative products, whether plant‐based or cell‐based, is growing fast and the food industry is facing a new challenge of creating innovative, nutritious, accessible, and natural dairy‐free cheese alternatives. The market demand for these products is continuing to increase owing to more people choosing to reduce or eliminate meat and dairy products from their diet for health, environmental sustainability, and/or ethical reasons. This review investigates the current status of dairy product alternatives. Legume proteins have good technological properties and are cheap, which gives them a strong commercial potential to be used in plant‐based cheese‐like products. However, few legume proteins have been explored in the formulation, development, and manufacture of a fully dairy‐free cheese because of their undesirable properties: heat stable anti‐nutritional factors and a beany flavor. These can be alleviated by novel or traditional and economical techniques. The improvement and diversification of the formulation of legume‐based cheese alternatives is strongly suggested as a low‐cost step towards more sustainable food chains. © 2021 The Authors. *Journal of The Science of Food and Agriculture* published by John Wiley & Sons Ltd on behalf of Society of Chemical Industry.

## INTRODUCTION

Today, the dairy industry is strongly engaged in developing new lines of innovative products, responding to the needs of those who adopt particular lifestyles such as the current widespread trends of strict vegetarianism, flexitarianism, and veganism. They are attracting the interest of dairy producers who are fully aware of the risk of losing them as consumers. The preparation of dairy products suitable for vegetarians is relatively easy, and it involves using vegetable rennet, such as that obtained from cardoon thistle, artichokes, Sodom apples, and fig tree latex, instead of animal rennet.^1^, ^2^, ^3^ However, only plant‐based ingredients are needed to create products suitable for vegans, who totally refuse any animal‐derived ingredients.

The introduction of vegan foods into the marketplace has made tremendous strides in recent years. Plant‐based cheese alternative (PBCA) is one of the many new emerging totally dairy‐free products responding to the requirements of people who choose to predominantly eat plant‐based (PB) food.^4^, ^5^ In 2016, the global market value of vegan cheese amounted to approximately 2.06 billion US dollars and this is predicted to increase to 3.90 billion dollars by 2024^6^ while sales of vegan cheese in the USA increased by 43% from 2009 to 2018.^7^ Plant‐based cheese alternative might also fit into the diets of people with special dietary needs such as those with cow milk allergy or lactose intolerance, and those with concerns about cow milk hormones.^8^ Consumer interest in these products is growing fast and is amplified by the large number of videos and recipes shared on social media of home‐made vegan cheese using legumes or nuts as basic ingredients blended with commercial fermented yeast and salt. Unflavored coconut oil is the main oil used, and for a desired meltability and stretchability texture, tapioca flour is usually added due to its viscoelastic and stretchy properties.^9^ Plant‐based cheese alternatives are perceived to be healthier than the original dairy versions as they have no lactose and no cholesterol.^10^, ^11^ However, Demmer *et al*. (2016)^12^ showed that the saturated fatty acids of a non‐dairy cheese alternatives containing palm oil increase blood pro‐inflammatory markers more than the saturated fatty acids of a dairy cheese.

In 2017, the European Union prohibited the terminologies ‘milk’, ‘cheese’, ‘butter’, and ‘yoghurt’ for non‐ dairy products^13^ and in 2018, the mandatory product labels ‘non‐vegetarian’, ‘vegetarian’, and ‘vegan’ were approved by the European Commission to support consumers following a PB diet to identify appropriate food products.^7^ Dairy product alternatives include plant‐based and cell‐based alternatives.^14^ Recently, attempts have been made to manufacture PB milk alternatives (fully or partially) from legumes, seeds, nuts, cereals, and pseudo‐cereals, like those derived from soybeans.^8^, ^11^, ^15^, ^16^ For cheese alternatives the range of plants tried is narrower. The main PB‐derived proteins used today are soy and nuts. Peanuts, cashews, macadamias, and almonds are usually used for nut cheese making.^17^ However, nuts are relatively expensive compared with the price of beans and cereals. As a result, the nut content (less than 5%) and consequently that of protein (less than 0.2 g) in the final product is low. Soy proteins are cheap and possess good functional properties; however, the consumption of soybeans and derivative products is limited because of their potential allergenicity and the concerns that some people have over genetically modified (GMO) soybeans.^18^

Legumes are considered to be a valuable source of potentially functional ingredients and a remarkable shift towards the increased consumption of legume proteins has been noticed in the past decade.^19^, ^20^ The last few years have been characterized by a growing number of published papers, as reported in the Web of Science database, addressing the themes ‘plant‐based cheese’ or ‘dairy‐free cheese’ or ‘vegan cheese’, ‘tofu’, ‘legume’, and specific named pulse proteins. They are considered from many perspectives: nutrition, technological properties, environmental impact, and food production. A systematic review of the scientific literature published after the year 2000 using ‘plant‐based cheese’, ‘dairy‐free cheese’ and ‘vegan cheese’ as search terms resulted in the identification of about 61, 8, and 31 scientific papers, respectively, while the term ‘tofu’ resulted in 1700 papers and ‘legume proteins’ resulted in 9955 papers. The highest number of publications of plant‐based, vegan and dairy‐free cheese was in the year 2020.

Today, one of the most critical challenges in the cheese industry is the design and development of safe products with high nutritional and functional characteristics using clean label ingredients that meet consumer expectations.^21^, ^22^ The purpose of this review is therefore to describe the current status of dairy cheese alternatives and to emphasize the role of legumes as valuable and low‐cost sources of proteins for consideration in these products.

## DAIRY PRODUCTS ALTERNATIVES: INNOVATIONS AND CONSUMERS' APPROACH

The meat‐free and dairy‐free food industry still has difficulties in delivering the right sensory experience and in mimicking the texture and flavor of the original product.^21^, ^23^ Among the dairy product alternatives, cheese remains the biggest obstacle for people considering going vegan. According to The Food and Health Survey, the taste and flavor of food play the major role in the consumers intention for purchasing.^24^ The PBCA industry has not yet managed to replicate cheese meltability and stretchability and most PBCAs in the market have a chalky, pasty, plastic‐like texture. Plant proteins have a higher molecular weight and different functional properties from milk casein and consequently it is hard to imitate the texture of cheese. The easiest cheeses to mimic are those with a spreadable and creamy texture such as feta, ricotta, or cottage cheese, as well as those with a strong flavor – e.g., spicy and smoky products, covering the flavor of the plant source.^25^ A second and more valuable approach would be to enjoy and accept the flavor of plant‐based ingredients and to consider the dairy product alternatives as innovative food to enlarge the range of vegan products. In fact, focusing on improving the resemblances (flavor, aroma, and physical appearance) between dairy food and the alternatives is a limitation that narrows the cheese alternatives market and make the protein transition from animal to plant more difficult.^24^

Today, consumers are more conscious about functional food and the adverse health issues associated with synthetic ingredients or food loaded with fat, sugar, and salt. As a result, they are asking for new vegan products with a high nutritional profile containing few and natural ingredients. They are mainly concerned about the protein content, and they are attracted by products made from legumes or nuts and fortified with calcium (as calcium salts) and vitamin B_12_. However, most of the commercial PBCAs found in the market do not respond to the consumers' needs, as they are mainly coconut‐oil based (74%), or nut based (10%) (mainly almonds and cashew).^26^ The market statistics and findings contradict the scientific literature, where PBCAs from soy proteins have been investigated most. The coconut‐oil‐based PBCAs contain a mix of starches; typically, a combination of native and modified potato and/or corn starch. The modified starch is another undesirable ingredient for many consumers.^26^ The dairy products category plays an important role in the diet of most people owing to their high content of calcium, proteins, and vitamins (especially the B complex).^27^ Plant‐based cheese alternatives have a lower nutritional value, e.g., calcium and protein content, than conventional dairy cheeses. Generally, 50% of commercial PB milk alternatives contain little to no protein (<0.5%).^28^ As a result, the development of cheese alternatives with a comparable protein content to dairy cheese would be a huge breakthrough in this sector. Legumes could be a better ingredient for PB dairy alternatives than any other plants thanks to their high protein content, almost twice, than whole grain cereals and pseudo‐cereals and their low cost compared to that of nuts. Legumes are poor in sulfur‐containing amino acids such as tryptophan, cysteine and methionine but are rich in lysine content while the composition of amino acids in cereals is vice‐versa. Consequently, legume proteins complement those of cereals and a mix of both might equilibrate the anabolic properties of PB protein intake.^29^, ^30^, ^31^

Despite their importance in human nutrition, pulses have been neglected in modern cuisine, for different reasons, including, but not limited to, the prolonged cooking time, lower protein content compared to meat and dairy food products, and the presence of anti‐nutrient compounds. However, legumes reappeared in the last decade, gaining considerable popularity among many consumers following increased awareness of the animal welfare, environmental sustainability, and healthy features of food.^5^, ^32^

Today, food specialists are increasingly introducing novel food to consumers. The protein base transition in the diet is changing rapidly. The first transition was from animal protein to plant protein while the second transition is to lab‐grown protein. Lab‐made dairy proteins and microalgae proteins are the latest inventions in dairy‐product alternatives. The former are based on an innovative technique that imitates the sensorial and physical experience of milk, yet, the cheese made is vegan, lactose‐free, and cholesterol free. It can be also called ‘*in vitro*’, ‘cultured’, ‘synthetic’, ‘clean’, and ‘cell’ agriculture. It involves converting the amino acids of the four main caseins and two whey proteins to DNA sequence and mixing them with a yeast population in a bioreactor under controlled conditions, mimicking the milk production system of mammals.^33^ According to Bryant and Barnett (2020),^34^ cultured meat and milk are among the future protein sources that the food industry will witness. Today, there is no commercial lab‐grown milk on the market, while prototypes of ice cream and yogurt have already been created, which suggests that the creation of cheese prototypes could be next.^14^ Studies of consumers' acceptance of, and willingness to try, cultured meat showed a higher rate of acceptance in the USA than in Europe, and in the Netherlands and Finland than in the UK, Spain, and Poland. Studies in Italy and Holland reported that more than 50% of the people included in the study are willing to try cultured meat.^35^

The single‐celled marine microalgae technique is the ultimate innovation to create new food products and to broaden the vegan food choices. It is the third biggest investment in the alternative protein industry. Producing microalgae‐based proteins requires less land than producing animal and plant proteins. A company in Singapore has produced the first milk from microalgae protein. It has created a strain of marine microalgae that could be mass‐cultivated under controlled conditions, grown on food waste from breweries, tofu makers and sugar refineries, and harvested in only 3 days.^36^, ^37^ Microbes produce protein (bulk protein) through lab biomass fermentation, and this is considered a more sustainable technique than plant protein production or lab cultured milk. Consumers acceptance of lab‐grown food is still under investigation. Consumers who doubt science and have food neophobia are less likely to accept cell‐based meat and milk alternatives. Lab‐grown food has not yet been defined legally and is sometimes not considered as a real food.^14^ The technical feasibility of producing large quantities of affordable lab‐grown meat successfully is another challenge. Finally, the cost of these foods will play a major role in the success of this new market. Although tofu and plant‐based cheese might not be attractive enough to consumers any more, legumes are still the safest and cheapest proteins to be used for dairy‐free cheese alternatives.^13^, ^38^ However, in general, all PBCAs are more expensive than cow cheese, with nut‐based cheese alternatives being more than three times more expensive than the other plant‐based ones.^26^ Usually the price of PBCA made from legumes does not mirror the price of its ingredients, which are usually cheaper than the dairy ingredients. This is because it is an innovative product produced on a small scale and its marketing is limited to a specific category of people – the vegans. We believe that legume‐based products should not be assigned to the vegan section, usually visited only by vegan people, in the supermarkets but they should be a food option to all consumers concerned about health and in continuing demand for novel and natural functional food free from synthetic additives.

## LEGUMES; COMPOSITION AND PROCESSING

Legumes belong to the *Fabaceae* family, and include, as major types, the common bean (*Phaseolus vulgaris*), the fava bean (*Vicia faba* L.), the soybean (*Glycine max* L. Merr.), the pea (*Pisum sativum* L.), the cowpea and the black‐eyed pea (*Vigna unguiculata* ssp. *unguiculata*), the pigeon pea (*Cajanus cajan* L. Millsp.), chickpea (*Cicer arietinum* L.), lupin (*Lupinus albus* L.), lentil (*Lens culinaris* Medik.), and peanut (*Arachis hypogaea* L.).^39^, ^40^ They have been a part of European diets for centuries,^41^ and are considered the major protein source in the traditional cuisine of the Mediterranean region.^42^, ^43^ These low‐cost seeds are considered the ‘meat of the poor’ and are a staple food of the low‐income communities in developing and underdeveloped countries.^44^

Legumes are rich in proteins of high biological value, carbohydrates, minerals (e.g., calcium and iron), vitamins (e.g., thiamin, and niacin) and bioactive compounds, and have low fat content. They are a low glycemic food (GI 31) because of their high dietary fiber, oligosaccharides, slowly digestible starch, and resistant starch content.^45^, ^46^, ^47^ Legumes have been shown to possess anti‐microbial, anti‐oxidant and anti‐inflammatory potentials.^40^ A high intake of legumes is associated with a low risk of metabolic syndrome.^48^, ^49^

Legumes provide 14.9–52.0 g/100 g wet basis (w.b.) of protein composed of the salt extractable storage proteins, and the globulins (>50%), further divided into 11S and 7S globulin subunits (GS), albumin, prolamin, glutelin, and residual proteins. Lupin and soybeans share a higher protein content than other legumes,^50^ and soybeans have the highest grain globulin concentration (Table 1). The latter, together with the ratio of 11S to 7S globulin subunits, are the key indicators of the functional properties of the proteins and their values differ depending on the legume plant sources and varieties (Table 1). Legume dry fractionation is a sustainable technique that has been shown to increase the grain protein percentage considerably.^55^, ^56^ Schutyser *et al*. (2015),^55^ Xing *et al*. (2020)^57^ and De Angelis *et al*. (2021)^56^ showed that the chickpea protein content could be increased from 21.6 g/100 g to 46.5 g/100 g in the protein‐enriched fraction. A disadvantage of dry fractionation, in contrast with protein isolation and concentration techniques, is that the anti‐nutritional factors (ANFs) are not eliminated and remain in the dry‐enriched fractions.^56^

**Table 1:** The percentage of globulin fraction in the total grain proteins, the denomination of globulin subunits 11S and 7S, the ratio of globulin subunits 11S over 7S and the protein digestibility‐corrected amino acid scores (PDCAAS) of chickpea, lentil, lupin, pea and, soybean.^51^, ^52^, ^53^, ^54^

Legume	Globulin (% of total proteins)	11S and 7S subunit denomination	11S/7S ratio	PDCAAS
Chickpea	60	Legumin and vicilin	1.60–3.70	0.59–0.82
Lentil	80	Legumin and vicilin	0.49–0.70	0.50–0.70
Lupin	85	*α*‐conglutin and *β*‐conglutin	0.77	0.80
Pea	60	Legumin and vicilin	0.50–4.20	0.79
Soybean	90	Glycinin and β‐conglycinin	0.6 0–3.00	0.90

### Grain chemical composition and health challenges

Legumes are strongly affected by challenges with digestibility mainly due to the presence of ANFs in the grain and the heat‐resistance property of their grain proteins. The protein digestibility‐corrected amino acid scores (PDCAAS) of unprocessed legume products are generally in the range of 0.40 to 0.70 (Table 1), which is not comparable with animal‐derived proteins except for lupin (0.8) and soybean (0.9).^58^, ^59^ Although heat treatment partially or totally inactivates the main ANFs, it appears to have remarkably little effect on the digestibility of some legumes. In pea, an improvement of only 10% of the *in vitro* protein digestibility was found after heating. Lentil protein was shown to be digestible *in vivo* when only detached from the seeds.^60^, ^61^ Lately, high hydrostatic pressure and legume extrusion have improved protein functionality and digestibility.^22^, ^62^

Trials of the application of diverse legumes in total dairy‐free cheese are limited due to the presence of the intrinsic beany flavor, which is mainly due to the activity of lipoxygenase (LOX) on unsaturated fatty acids (FA), producing hexanal, and the secondary plant metabolite ANFs, responsible for reduced nutrient digestibility, gastro‐intestinal distress, and allergic reactions experienced by some people.^63^, ^64^, ^65^ These ANFs include, phytic acids, tannins, alkaloids, saponins, phenolics, the undigestible carbohydrates *α*‐galactosides (raffinose, stachyose, ciceritol and verbascose), isoflavones, and the anti‐nutritional proteinaceous compounds, e.g., trypsin inhibitors, chymotrypsin, lectins, and antifungal peptides.^66^, ^67^ However, knowledge has been gained to overcome these problematic properties and diverse solutions were reported:^11^ (i) breeding varieties devoid of lipoxygenases (LOX), e.g. the modern sweet lupin, which is free from the bitter taste;^68^ (ii) economic and/or traditional treatments before grinding or cooking; dehulling, seed germination, alkaline (NaHCO_3_) soaking, blanching and, dry heating (roasting at 180 to 200 °C for 15 to 20 min proved to reduce the beany flavor and the ANFs);^69^ (iii) infrared heating of seed or micronization^69^, ^70^ (Table 2); (iv) removing short‐chain FAs, sterols, and sulfur compounds using a vacuum at high temperature;^28^ (v) the Cornell hot grinding method (in boiling water) to inactivate LOX (slurry kept at 80 °C for 10 min), which can be combined with a two‐phase ultra‐high‐temperature (UHT) processing (vacuum evaporation at 50 kPa);^28^ (vi) steam flashing to strip volatiles; (vii) use of defatted flour, protein isolates (PI) and concentrates (PC);^28^ (viii) fermentation or enzymatic treatment of seeds or the slurry, which might or might not be combined with high‐temperature pretreatment;^80^, ^81^ (ix) innovative non‐thermal processing techniques such as high hydrostatic pressure (HHP), high and ultra‐high pressure homogenization (HPH and UHPH), pulsed electric field (PEF),^11^, ^82^ ultrasonication,^82^ and radio frequency^78^ (Table 2); (x) addition of food natural or synthetic additives (gums and flavors) to mask the ‘off’ flavor;^28^ and (xi) milk deodorization to remove the ‘off’ flavor.^83^

**Table 2:** Effects of processing technologies on beany flavor and anti‐nutritional factors of various legumes and legumes‐based products

Technique	Legumes	Treatment parameters	Inference	References
Micronization or infrared treatment	Lentils	Previously tempered to 33 g/100 g moisture for 16 h, heating to up to 138 °C internal temp.	Decreased the phytic acid level, improved digestibility, and reduced trypsin inhibitors	70
	Cowpea, kidney bean and pea	Previously tempered to 24 g/100 g, heating at 90 °C using tubular quartz infrared lamp (115 V) for 2.5 min for cowpea and pea and 3 min for kidney beans	Reduced the phytic acid level, oligosaccharides, and trypsin inhibitors	69
High hydrostatic pressure (HHP)	Soymilk enriched with calcium	614 MPa, 85.5 °C, and 8.53 mmol Ca L^–1^	Inhibited trypsin inhibitors and lipoxygenase enzymes	71
High and ultra‐high pressure homogenization (HPH) and (UHPH)	Soy milk	200 MPa, 55–75 °C and thermal pasteurization at 90 °C for 30 s	Reduced hydroperoxide index values and trypsin activity	72, 73
Pulsed electric field (PEF)	Soybean LOX	20–42 kV cm^–1^; 2 μs pulse width; 1036 μs treatment time	Inactivated LOX (88%) at 42 kV cm^–1^ when treated for 1036 μs.	74
	Soybean LOX	20–40 kV cm^–1^; 25–100 μs; 23, 35, 50 °C	Inactivated LOX (85%) at the highest processing conditions	75
	Pea LOX	2.5–20 kV cm^–1^; 1 μs pulse width; 100–400 pulses	No inactivation	76
Ultrasonication	Soy milk	20 kHz, 15–20 min, 600 W	Decreased trypsin inhibitors (52%) after 16 min of the treatment	77
Radio frequency (RF)	Soybean	27.12 MHz and the electrode gap was set at 45 mm during RF heating period. Soybeans were stored at 30 °C and heated for different time from 30 to 180 s at 2.1 kW, and then were maintained at those temperatures for 120 s. Technique was compared with conventional hot‐air‐heating at 132 °C for different times	Reduced LOX (95.2%), urease (93.4%) and trypsin inhibitor (89.4%) activities. Compared with the conventional thermal treatment, RF heating efficiently inactivated ANFs with a shorter time and a lower treatment temperature	78
Combined high temperature pre‐treatment heating and enzymatic hydrolysis	Soybean isolates (SPI)	Temperature was increased to 121 °C at a heating rate of 17 °C min^–1^. After heating, the temperature was held for 3 min at 121 °C and cooled for 2 h at room temperature. SPI was then hydrolyzed by *Bacillus amyloliquefaciens* and *Bacillus licheniformis* (1.5 AU‐NH g^–1^).	Reduced LOX activity and some volatile compounds e.g., hexanol, hexanal, and pentanol	79

Other disadvantages that legumes could impart to the final product are the undesirable color (greenish, grayish, or brownish) and/or texture (chalky or sandy).^8^ Many PB milks labels show the use of additives and artificial flavorings to improve the taste and overall sensory quality of the products. However, additives are not well accepted by many consumers and are perceived as ‘unnatural’ products.^84^

### Legume protein isolates and concentrates

Protein isolates (PIs) (protein content higher than 80%) and concentrates (PCs) (protein content 50–80%) from legumes are free of color, flavors, odors, and ANFs, and consequently could be a good option to be used in innovative PB products.^22^, ^85^ Protein isolates are prepared from defatted and dehulled beans and undergo more processing steps than protein concentrates.^86^ A flour‐defatting process could be performed using a solvent or an eco‐friendly method, i.e., pressurized CO_2_ extraction.^87^ The legume protein isolates and concentrates are first solubilized at pH 8–9 and then extracted and isolated by isoelectric precipitation (around pH 4.5). Microfiltration or ultrafiltration can be further adopted to increase the amount of extracted proteins.^45^, ^83^, ^88^, ^89^ Microfiltration, which is considered a non‐thermal sterilization technique, could also serve to eliminate the microorganisms and improve shelf life.^90^ However, the loss of albumins occurring during the protein isolation process may be detrimental to the foaming properties of the legume‐derived milk.^91^ Protein extracts are stored and used in the food industry in a powder form. They are dried using the lyophilization (freeze dried) or convective drying techniques. Generally, the latter is used in the commercial production owing to its lower cost compared to the other technique.^92^ The insoluble fiber residue, and the acid‐soluble ‘whey fraction’ collected can be dried and utilized as improver of food shelf stability.^93^

### Fermentation

Fermentation is an old technique used principally for the preservation and enhancement of micronutrient availability and amelioration of the sensorial properties and health benefits by promoting intestinal health and immune system, of countless food products.^94^ Legume‐based cheese alternatives can be produced with or without fermentation. The main starters used are lactic acid bacteria (LAB), bacilli, and yeasts (e.g., *Saccharomyces*).^95^, ^96^ Beany flavor is alleviated through enzymatic hydrolysis, and the phytate content is reduced owing to the endogenous phytase of the seeds, and of the added yeast and other useful microorganisms while protein digestibility is improved.^97^ However, Yousseef *et al*. (2016)^98^ found that lactic acid fermentation was not efficient in improving the negative compounds associated with pea proteins. Usually, a blend of diverse strains is more used and beneficial than a mono‐culture.^99^ The fermentation of cowpeas using a mix of *Lactobacillus acidophilus* and *Lactobacillus plantarum* cultures was effective in alleviating the phytic acids and trypsin inhibitors.^100^ A mix composed of six to nine strains, including yeasts (*Geotrichum candidum, Kluyveromyces marxianus*, and *Candida catenulata*), lactic acid bacteria (*Lactococcus lactis, L. plantarum* and *Lactobacillus casei*) and other bacteria (*Hafnia alvei*) effectively fermented the partially substituted dairy milk with pea milk and triggered the formation of banana and apricot aromas.^73^, ^101^ Fermentation of faba bean flour enriched with protein by air classification leads to a reduction of vicine and convince by more than 90% and of trypsin inhibitors by 86%.^81^ For soybeans, a combination of *Streptococcus thermophilus* CCRC 14085 and *Bifidobacterium infantis* CCRC 14603 lowered the phytic acid (80%) and saponin (30%) content,^102^ while the mix of *Streptococcus boulardii* and *L. plantarum* B4495 improved considerably the calcium bioavailability when compared to a mono‐culture fermentation.^103^ Red bean fermentation with *Bacillus subtilis* had a higher antioxidant activity than the non‐fermented product.^104^ A combination of *L. plantarum* L1047 and *Pediococcus pentosaceus* P113 was efficient in alleviating the beany flavor in lupin protein food derivatives.^105^ A mix of *S. thermophilus, Lactobacillus bulgaricus* and *L. acidophilus* was effectively used in the fermentation of chickpea‐based products.^106^ Fermented cashew nuts with *Pediococcus* and *Weissella* genera, obtained through a quinoa starter inoculum named ‘Rejuvelac’ starter culture, had a very low allergenicity.^107^

## FORMULATION OF PLANT‐BASED CHEESE ALTERNATIVES

The technological and sensory quality of a cheese depends on the viscosity, emulsification, gelation, and meltability of the gel matrix formed during coagulation. In cheese production, these are controlled by the interaction of hydrolyzed caseins with melted milk fat.^108^ Dairy cheese can be achieved by a rennet‐induced (enzymatic) or acid‐induced (acidification) coagulation. When milk coagulates under rennet and normal conditions of pH and protein content, the viscosity does not increase until the enzymatic phase is mostly complete. Plant‐based cheese making follows a proper regime according to the characteristics of plant proteins. The first step is the plant‐based milk production, which is the water extraction of plant material. Plant‐based slurry is a colloidal system, and it is difficult to obtain a stable homogenic product with a long shelf‐life. The instability of the milk results in a sandy, granular texture, which is not creamy, caused by the deposit of solid and insoluble large particles.^25^ Innovative processing technologies are used to preserve the nutritional profile and to protect the physical stability by decreasing particle size, reducing viscosity, and inactivating microorganisms and enzymes in the final product, and to minimize the need for additives such as hydrocolloids and emulsifiers.^11^, ^109^ The novel technologies applied to plant‐based milk substitutes are ultrasound, high‐intensity ultrasound irradiation, PEF, ohmic heating, HPH and UHPH.^11^, ^82^, ^90^ For a detailed description of the effect of innovative processing technologies on various plant‐based products, see the extensive reviews of Munekata *et al*. (2020),^11^ Aydar *et al*. (2020)^90^ and Vanga *et al*. (2021).^82^

It is necessary to add starches and/or hydrocolloids to ameliorate the texture of a cheese matrix; however, producers of PBCA must always consider the environmental costs of all the added ingredients.^7^ As for the process, pulse milk prepared for PBCA production could be extracted from the blended whole seed, the flour, the protein isolates, concentrates or hydrolysates. Usually, PB milk is pasteurized before the cheese processing, which makes the cheese‐like product appropriate for all stages of the life cycle, including pregnancy, lactation, infancy, childhood, adolescence, older adulthood, and for athletes.

### Plant protein

Legume proteins are gluten free. When processed, they control the physiochemical properties of the gel formed and consequently the technological performance of the end product.^110^ They determine the water‐holding capacity (WHC) and solubility, the emulsion properties – i.e., emulsion ability (EA) and stability (ES) – the foaming capacity, flavor binding, viscosity, and gelling capacity. Studies on chickpea, lentil, pea, and lupine proteins have proved their good EA, ES, and foam stabilization capacity and they are therefore believed to be a potential alternative to meat and dairy proteins in food.^45^, ^95^, ^110^, ^111^, ^112^, ^113^, ^114^

A blend of different legume sources could also be used with the aim of attaining higher technological and nutritional attributes. The addition of gluten to PBCAs is common and has a dual purpose: to increase the protein content in the final product and to give the stretchability or the fibrous effect of the stretchy cheeses like Italian Mozzarella and Stracciatella.^115^ Given the similarity among the protein fractions of the different legume sources, similar functions and potential applications are expected.^116^ The protein functionality is affected by the plant source, genotype, conditions influencing the protein denaturation (pH, ionic strength, presence of free sulphydryl or disulphyde group) and the cooking parameters (temperature, heating time, and rate of cooling).^31^, ^112^ The WHC, EA, and ES are mainly regulated by the protein concentration and composition (proportions of the 7S and 11S globulins) and, to a lesser extent, by the oil fraction and environmental conditions (pH and ionic strength).^117^, ^118^, ^119^, ^120^ In fact, a positive correlation between solubility and emulsifying capacity was found in pea protein isolate.^121^ Can Karaca *et al*. (2011)^112^ showed that, at pH 7, lentils have a higher emulsion capacity than chickpeas, fava beans, peas, and lupins while, at the isoelectric point, lentils and chickpeas have a similar creaming ability, EA, and ES to soybeans. Although legume proteins are considered a good potential ingredient for novel food, some research areas on the technological characteristics of legumes are still unexplored. Among the most commonly studied plant proteins are pea and soy proteins. Soy proteins are incorporated in a broad‐spectrum of food products thanks to their ability to ameliorate the texture of the products^122^ and are usually used as a control reference when studying proteins from other legume sources. In terms of functionality, according to Tulbek *et al*. (2017)^123^ gel made from pea protein isolate is weaker than that of soybean, but it can be improved by applying enzymatic treatment, e.g., transglutaminase. However, pea protein isolate is a better emulsifier and foaming agent at pH 7 compared to soy protein isolate. According to Nivala *et al*. (2021),^124^ fava protein isolates have higher water and oil absorption capacities but lower foaming capacity and stability than pea and soybean isolates. Lentil, pea and lupine proteins retain a weaker gelling capacity than chickpea and soy proteins as measured by the least gelling capacity index (LGC).^45^, ^113^, ^115^ The latter could be improved by the fractionation technique.^125^ The gel‐formation ability of legume protein is crucial for its use in cheese‐like processing. The interaction of the globulin storage proteins generates soluble aggregates. In the case of soybeans and lentils, the gelation rate obtained by the heated‐storage 11S globulin proteins is slower than that of 7S proteins, and the gelation time is longer than that of 7S. The gel of 11S globulins is turbid and hard, whereas that of 7S is susceptible to rupture and transparent.^13^, ^126^, ^127^ Cai *et al*. (2002)^128^ showed that the curd of soybeans, chickpeas, and fava beans had greater textural characteristics (hardness, springiness, and cohesiveness) than that of lentils, smooth peas, and mung beans, owing to their higher 11S over 7S globulins ratio. The proportions of the globulin subunits vary among genotypes. Varieties with higher 11S over 7S ratio form a harder gel, more cohesive and gummier and, as a result, a tougher cheese. Consequently, the gel behavior of soybean depends on the variety used and selecting or breeding varieties with improved gelling properties is possible.^129^ The rheological properties of the gel, as well as the foaming and emulsification abilities, could be impaired by heat treatment and Wang *et al*. (2020)^130^ showed, using chickpea protein isolate, that they can be improved by high intensity ultrasound.^114^, ^130^ Xu *et al*. (2021)^131^ compared the functional properties of protein isolates and hydrolysates of pigeon pea, lentil, and chickpea when hydrolyzed by alcalase and bromelain and showed that the water absorption and oil binding capacities of the three legume proteins were improved by bromelain application.

### Vegetable oil

Vegetable oil or fat, a cheap substitute for milk fat, is an essential ingredient in the PBCA formulation as it improves the texture, especially the melting properties and mouthfeel, of the final product, and makes it more similar to dairy cheese.^26^ It is added before the coagulation or fermentation process. Unflavored coconut oil is the main oil used today in the cheese‐like industry, owing to its high fat content in saturated fatty acids (80–90%) and consequently high melting point, followed by palm (51.4%) and sunflower oils (12.6%). Rapeseed, soybean, and safflower oils can also be found in the vegan cheese industry.^23^, ^26^ Mattice and Marangoni (2020)^9^ blended coconut oil (75%) with high oleic sunflower oil (25%) to imitate the ratio saturated over unsaturated fat found in cow milk. Usually, a partially hydrogenated oil is used to make semi‐hard cheese while a hydrogenated oil is used to make a hard cheese.^26^ The melting profile of the oil is associated to the mouthfeel and hardness of the final product. Fat replacer, e.g. maltodextrin, can also be found in PBCAs. The addition of a vegetable oil rich in omega 3, e.g., flaxseed, rapeseed, and soybean, could be beneficial for the fortification of the cheese substitutes with EPA and DHA, the omega 3 long‐chain polyunsaturated fatty acids compounds, responsible of many physiological benefits.^132^

### Coagulants and food thickeners

In PBCA production, a single or a mix of two or more coagulants and/or food thickeners can be added to achieve the desired texture of the end product. Coagulation behavior depends on the coagulant type, its concentration and time of application, the plant protein source and variety, and the cooking conditions, such as temperature of the milk and pH. Coagulant can be applied with or without heating, although this latter was shown to ameliorate the formation of gel in soy cheese making. Stirring for a short time after its addition was shown to significantly improve the curd yield.^103^, ^127^, ^133^

The coagulants reported in the literature and used in legume‐based cheese processing, and mainly in tofu making, are categorized into: (i) Acid, e.g. lactic acid, tartaric acid, malic acid, glucono‐*δ*‐lactone, citric acid. This is usually added at the concentration of 0.2 to 1% of the mixture and it acts by decreasing the pH to the isoelectric point of the protein.^127^, ^133^, ^134^, ^135^ (ii) Salts, e.g. calcium sulfate, calcium chloride, calcium acetate, calcium lactate, magnesium sulfate, magnesium chloride (which could impart a bitter taste), and trimagnesium citrate. They are added at a concentration of 0.4 to 0.5% of the mixture and act either by inducing a cationic salt bridge (a thermally induced cross‐linking between metal ions and plant protein), or a salting‐out effect (protein dehydration followed by heat denatured plant protein) or acting as an acid coagulant and, consequently, lowering the pH value to the isoelectric point of the protein.^121^, ^123^, ^136^ (iii) Enzymes e.g., Sodom apple extract (*Calotropis procera*), Roselle calyces (*Hibiscus sabdariffa*), papain, microbial transglutaminase (100 U/100 mL of plant milk).^127^, ^133^, ^135^, ^137^, ^138^ (iv) Cold, e.g. Hagfish slime hydrogel.^133^ (v) Natural coagulants, e.g., chitosan, viz. gooseberry (*Phyllanthus acidus*), tamarind (*Tamarindus indica* L.), lemon (*Citrus limonum*), garcinia (*Garcinia indica*), and passion fruit (*Passiflora edulis*).^135^, ^138^, ^139^, ^140^

Coagulants, and particularly organic acids, may influence minor components of PBCAs, such as vitamins, mineral salts, or polyphenols. For example, organic acids used for coagulation may enhance the absorption of iron. This effect is important in diets/foods rich in inhibitors, such as phytates or tannins. In particular, besides the known ascorbic acid (vitamin C), various other organic acids e.g., acetic, citric, lactic, malic, and tartaric acids may increase iron solubility, depending on pH, iron source, ligand, processing methods, and the food matrix. Furthermore, a synergistic effect has been reported for the combination of ascorbic acid with lactic acid.^141^

The absorption of vitamins can also be influenced by organic acids, which may have a negative effect on the absorption of folates. Organic acids, in fact, may influence the hydrolysis of polyglutamyl folates (which represent the majority of the total folate intake from a mixed of unfortified diet) to monoglutamate, needed for absorption by the proximal small intestine.^142^ This process is catalyzed by the glutamate carboxypeptidase II (GCPII) enzyme, having an optimum pH at 6–7, so lower pH values may result in the incomplete intestinal deconjugation of polyglutamyl folates. Organic acid ions (citrate, malate, ascorbate, and phytate), present in orange juice, have a combined inhibitory effect on the activity of GCPII.^143^

Organic acids also influence the level of polyphenols by means of their inhibitory effect on polyphenol oxidase (PPO), whose optimal pH ranges between 4 and 8 depending on the plant species.^144^ Organic acids may therefore prevent undesired enzymatic browning.

One or a mix of two food thickeners, hydrocolloids (such as agar, guar gum, xanthan, carrageenan, gum arabic, tragacanth gym, inulin, gelatin), or vegetable microfibers (such as oat microfiber and bamboo microfiber) could also be used.^32^, ^133^, ^140^ According to Saraco (2019),^26^ the most commonly used gum was carrageenan, mostly associated with guar gum, a galactomannan that exhibits thickening properties but cannot form gels. While Oat fiber was found to be the most commonly used plant fiber, mainly used for the production of hard and extra‐hard PBCAs.

Starch might also be used as a thickener and moisturizer in PBCAs. The main starch sources found in the literature are tapioca, rice, maize, pea fiber, and potato. Modified starch, of corn and potato, is used in commercial PBCAs, although this is deemed unhealthy.^26^ Products made from powder blends having a combination of tapioca starch, hydrocolloid, and pea protein with weight ratio of 7:2:1 demonstrated the best strand capacity and meltability.^145^ The increase in the starch content results in an increase in the rigidity and hardness and a decrease in the meltability of the final product.^146^ The soft cheese‐like alternative presents low proportions of starches (about 5%), whereas the hard type exhibits a higher amount (about 30%).^26^

### Other ingredients

Plant‐based cheese alternatives can have a smoked or sweet taste and can be eaten raw, cooked, or fried. For cheese seasonings, herbs, spices, and flavored salts can be added.^147^ Other minor ingredients, which are nevertheless critical for the technological and sensorial quality of the cheese, include chemical or natural antimicrobial agents added to improve the safety and shelf‐life of the product, salt (0.5 to 2% of the final product), and emulsifying agents such as genipin (a gardenia extracted novel natural crosslinking agent),^148^ lecithin, maltodextrin, and mono and diglycerides. Artificial flavoring additives labeled as ‘flavoring’ such as mozzarella, gouda, cheddar, and other cheese flavors are commonly used. For natural flavorings, the addition of vegetables, such as carrot puree or onion powder, was noted. Many PBCAs also contain yeast extract or nutritional yeast.^26^ Plant‐based diets are nutritionally inferior to the omnivorous diets and food processing and techniques used for the elimination of the beany flavor and ANFs contribute further to the deficiency in nutrients, so fortification agents are recommended.^28^ Probiotics (Lactobacilli and Bifidobacteria), vitamin D, calcium with an optimum calcium to phosphorus ratio (1.3:1), vitamin B_12_, iron, zinc, and omega 3 have been listed in the literature as critical and valuable fortifying agents.^7^, ^149^, ^150^, ^151^

## CONCLUSION

Cheese is an important food in human nutrition, and a dairy‐free product that is similar in texture and use to cheese is needed in the modern food market, although its technological properties should not be achieved by compromising the nutritional value of the end product. Many studies have been conducted on the technological and nutritional properties of the legumes‐based beverages; however, studies on legume‐based cheese alternatives are scarce. Consequently, further studies are required from many perspectives to widen the range of nutritious end products. They include technological research to alleviate ANFs using sustainable techniques, consumer liking and approval studies, and nutritional studies for fortification purposes and to find natural coagulants/thickeners and secondary ingredients. It is important to address these challenges in order to deliver the clean‐labeled and high‐quality cheese‐like products that the consumers are requesting.
